# Video Killed the Radio Star—A Meta‐Analysis on Video‐Based Coaching to Improve Surgical Skill

**DOI:** 10.1002/wjs.12494

**Published:** 2025-02-12

**Authors:** Steven Mark Anderson, Sarahlouise Gillanders, Anne Hickey, Gozie Offiah, Niall Davis

**Affiliations:** ^1^ Department of Surgical Affairs RCSI University of Medicine and Health Sciences Dublin Ireland

## Abstract

**Background:**

The surgical trainee of today completes their postgraduate training with significantly less exposure than their mentors. The enforced reduced working hours, along with other factors, have created a gap in surgical training. Video‐based coaching (VBC) provides an opportunity to improve the surgical technical skill without needing to increase surgical volume. The aim on this study is to investigate the effect of VBC on the surgical technical skill.

**Methods:**

A systematic review of randomized controlled trials was conducted in accordance with the Preferred Reporting Items for Systematic Reviews and Meta‐Analyses guidelines. Bias was assessed for using The Cochrane Collaboration's tool for assessing risk of bias. The study was prospectively registered in the Open Science Framework (https://osf.io/sp8rb). Multiple subgroup analyses and meta‐analyses were carried out, with results reported as standardized mean differences (SMDs) in performance scores and presented as forest plots.

**Results:**

A total of 15 studies comprising 382 participants were included in the final analysis. From these 15 studies, 201 participants received VBC following a range of surgical procedures. On meta‐analyses, the average performance scores for VBC were significantly higher than the controls (SMD 0.71, 95% CI 0.37, 1.04, *Z* = 4.15, and *p* < 0.0001) as were the average change scores from baseline to final performance (SMD 0.98 [95% CI 0.61, 1.35, *Z* = 5.19, and *p* < 0.0001]). Furthermore, the overwhelming majority of VBC participants across the studies found VBC to a be useful training tool.

**Conclusions:**

This review represents the most comprehensive assessment of the impact of VBC in surgery and demonstrates it to be an effective training tool in improving surgical technical skill acquisition. Training bodies around the world should now look at how best to formally integrate VBC into conventional surgical training.

## Graphical abstract

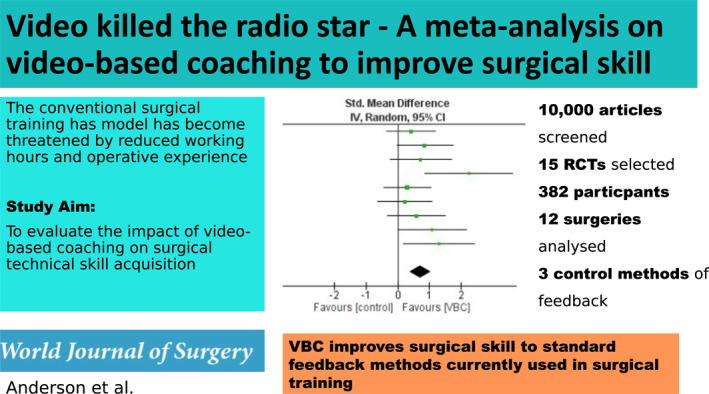

## 1 Introduction

Conventional surgical training is based on an apprenticeship model which became formalized by William Halsted and had long been successful due to its reliance on a close trainee–trainer relationship to facilitate intraoperative learning [1]. Many external pressures have strained this relationship to breaking point. Longer waiting lists have led to operating lists being overbooked, adding time pressure to each case and reducing training opportunities [2]. Furthermore, although reduced working hours are a positive initiative in medical education as a whole, an unhappy consequence is that the graduating surgeon of today does so with significantly less experience than their predecessors [3]. Therefore, it is clear that the conventional model of surgical training must now be supplemented with novel training techniques.

Although simulation training has gained popularity in recent years due to its ability to facilitate deliberate practice without concerns of patient safety, the significant cost associated with the development of a sophisticated simulation program limits its widespread availability [4, 5]. Video‐based coaching (VBC), in which a coach provides individualized feedback on a trainee's performance after reviewing video footage of their performance together, allows us to reproduce the conventional trainee–trainer relationship without the time constraints of the operating room and without the need for sophisticated simulation models. Therefore, it is unsurprising that there has been a growing interest in the use of VBC in surgical training. Although the perceived usefulness of VBC reported in the literature to date has been overwhelmingly positive, comparatively few studies have reported on its impact on technical performance [6]. Of the studies that have assessed technical performance, the majority are of small sample size with many failing to demonstrate a statistically significant difference [7, 8]. As a result, the use of VBC within surgery remains sparse and its role in future training unclear. The aim of this study is to perform a systematic review of the literature to evaluate the current evidence on how VBC influences surgical technical skill acquisition and to evaluate its role in surgical training.

## 2 Methods

### 2.1 Study Design and Search Strategy

A systematic literature search of Medline, Embase, EBSCOhost, Ovid, Scopus, and Web of Science was performed using the following search terms: “video*”, “film*”, “coach*”, “feedback*”, “mentor*”, “train*”, “teach*”, and “surgery*”. The following MeSH (medical subject headings) were also used: “videotape recording”, “mentoring”, and “surgical procedures, operative”. The references of all included studies were also reviewed to identify further eligible studies. Finally, the Google Scholar database was also used to identify ongoing research projects. The search strategy was predefined and listed within the study protocol, which was prospectively registered in the Open Science Framework (https://osf.io/sp8rb).

### 2.2 Eligibility Criteria

The study selection was limited to randomized controlled trials (RCTs) that compared VBC to a defined control group and included an objective assessment of the surgical technical skill as the primary outcome measure. Two authors (SA and SG) independently examined the entire search results for potential studies. Initially, duplicates were removed, titles were screened, followed by abstracts and ultimately full manuscripts. Disagreements on the study selection were resolved through open discussion with the senior author (ND). The full eligibility criteria can be found in the Supporting Information S1.

### 2.3 Data Extraction and Analysis

A critical appraisal of the included studies was performed using The Cochrane Collaboration's tool for assessing risk of bias [9]. The primary endpoint of interest was the mean change in technical skill performance scores. The secondary endpoints of interest were participants perceived usefulness of VBC and change in time to task completion (see Supporting Information S1). The two authors who performed the literature search also independently extracted the data and critically appraised the selected studies. Data on study characteristics (country of origin and study year), sample size, study population, intervention characteristics (number of coaching sessions, length of sessions, and coaching model), control methods of feedback, and performance scores were extracted.

### 2.4 Statistical Analysis and Data Reporting

Search results are reported in accordance with the Preferred Reporting Items for Systematic Reviews and Meta‐Analyses as outlined by the PRISMA 2020 Statement [10]. Statistical analyses were carried out by the Review Manager (RevMan–v.5.3.5 The Cochrane Collaboration 2012). The differences in effect size between the control and intervention groups of the included studies were compared and reported as standardized mean differences (SMDs), with 95% confidence intervals (CIs). Statistical heterogeneity was assessed by comparing the confidence intervals of the included studies and using the *I* ^2^ statistic. To best control for the heterogeneity seen, the meta‐analyses were performed using a random effects model of inverse variance. The test for overall effect is reported as Z scores, and a “*p*” value of less than 0.05 was considered statistically significant. Where data were missing, the corresponding authors were contacted and the data requested. Where there was no response, the missing data were imputed (see Supporting Information S1 for further description of the statistical analysis).

## 3 Results

### 3.1 Search Results and Study Characteristics

**FIGURE 1:**
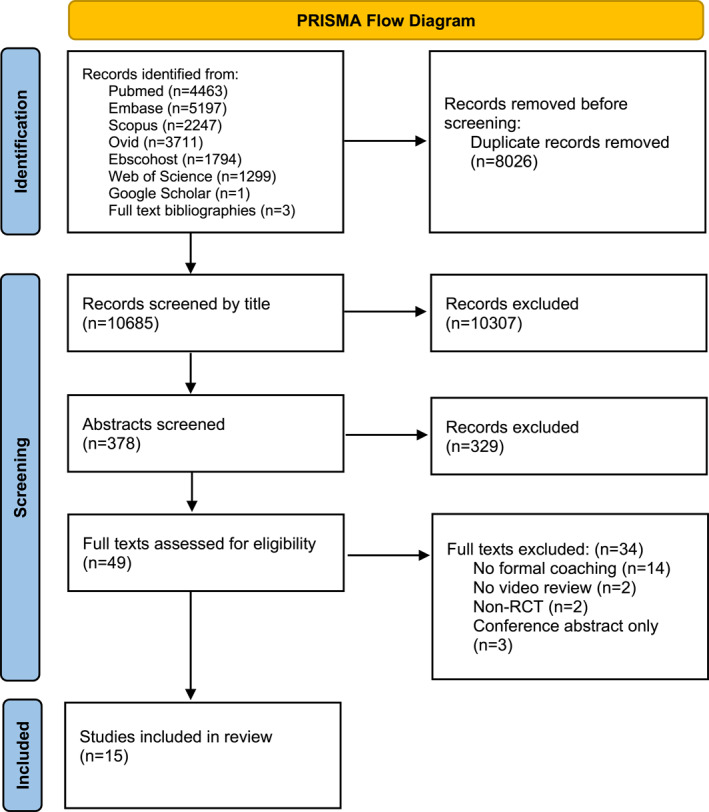
PRISMA flow diagram.

The initial search, following removal of duplicates, identified 10,685 articles. Following an initial title and abstract screen, 49 full texts were selected for review, of which, 34 were excluded for various reasons. The reasons for study exclusion are outlined in the PRISMA flow diagram (Figure 1). In total, 15 studies comprising 382 participants satisfied the predefined eligibility criteria and were included in the final review.

**FIGURE 2:**
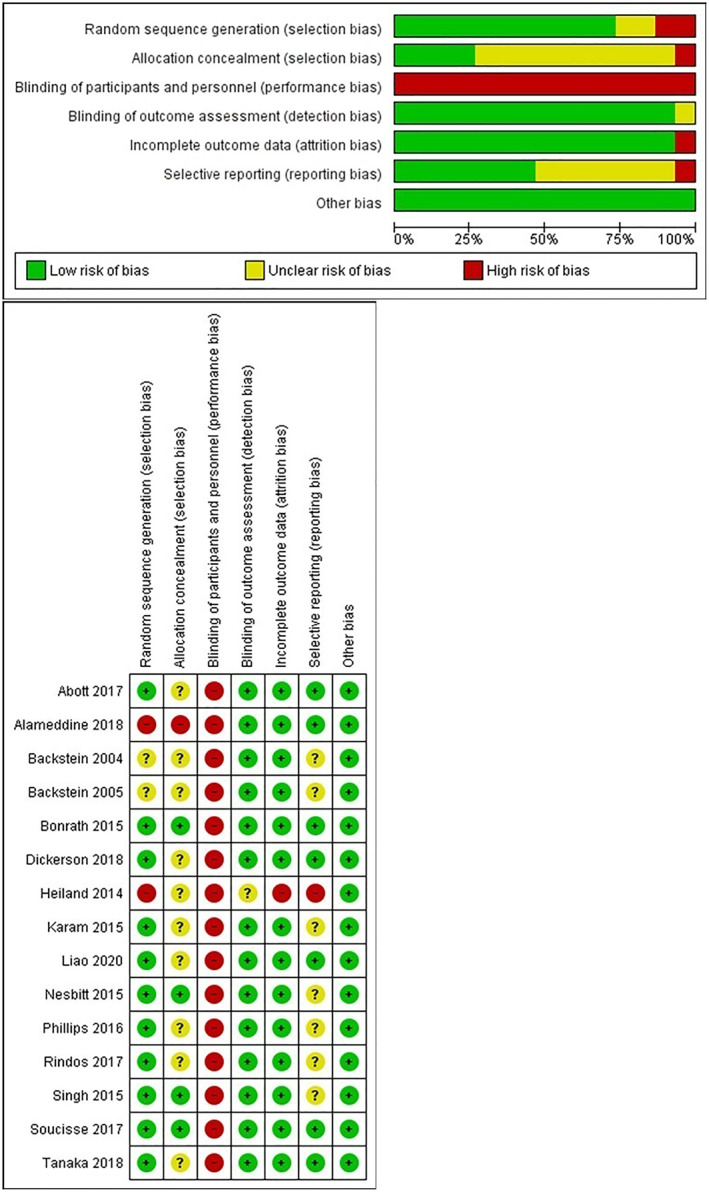
Critical appraisal.

The methodological quality of the included studies was generally good (Figure 2). All the studies scored a high risk of performance Bias as it is not possible to blind participants or personnel to VBC or control. However, with the exception of one study by Heiland et al., all of the remaining studies scored a low risk of bias across most categories [11].

### 3.2 Study Participants and Index Procedures

Of the 15 included studies, 10 assessed the impact of VBC on surgical residents across general surgery, orthopedic, neurosurgery, and gynecology training programs, whereas the remaining five studies included undergraduate trainees only. Only four studies included video footage of trainees performing in vivo surgeries performed during their residency program as the index procedure for both the VBC and objective assessment of the technical skill. The remaining 11 studies used procedures performed on benchtop models in a simulated setting. The index procedures are described further in Table 1.

**TABLE 1:** Study characteristics.

Author (year)	Participants			Procedure	
	Total no.	Year of residency	Surgical specialty	Type	Task
Backstein (2004)	29	1–5	Orthopedics	Simulation	Pinning and plating long bone fracture, cerclage wiring of olecranon fracture, Z‐plasty
Backstein (2005)	26	1	General surgery	Simulation	Vascular anastomosis
Heiland (2014)	12	1–5	Neurosurgery	Live surgery	Lumbar spine surgery
Bonrath (2015)	18	3–5	General surgery	Live surgery	Laparoscopic jejunojejunostomy
Nesbitt (2015)	32	Undergraduate	N/A	Simulation	Skin suturing on artificial skin
Karam (2015)	15	1–2	Orthopedics	Simulation	Open reduction and internal fixation of an intra‐articular tibial plafond fracture
Singh (2015)	20	Undergraduate	N/A	Simulation	Virtual reality and porcine laparoscopic cholecystectomy
Abbott (2017)	21	1	General surgery	Simulation	Laparoscopic knot‐tying
Phillips (2017)	71	Undergraduate	N/A	Simulation	Skin suturing on artificial skin
Rindos (2017)	20	1–4	Gynecology	Simulation	Laparoscopic suturing on a vaginal cuff model
Soucisse (2017)	28	1–4	General surgery	Simulation	Intestinal anastomosis on cadaveric dog bowel
Alameddine (2018)	16	Undergraduate	N/A	Simulation	Skin suturing on artificial skin
Tanaka (2018)	16	1–5	General surgery	Live surgery	Laparoscopic inguinal hernia repair
Dickerson (2019)	42	1–5	Orthopedics	Simulation	Distal tibia fracture reduction model
Liao (2020)	16	1–2	Not specified	Live surgery	Lap appendicectomy

### 3.3 Coaching Strategies

All the included studies employed coaching strategies that involved exposing the participants from the VBC intervention groups to a video of their own performance with feedback from an expert. However, the number of coaching sessions ranged from a single session to 10, the duration of each session ranged from 10 to 45 min, and the timing of each session varied from immediately following the procedure to 2 weeks following the procedure (Table 2). Similarly, the structure of the expert feedback delivered within the coaching sessions varied significantly across the studies. Fourteen of the studies used one‐to‐one feedback with the “coach” and participant reviewing the video of the procedure together, whereas Abbott et al. provided the participants in the VBC group with an edited video of their performance with a voiceover from a coach providing feedback [12]. Only three studies reported using a previously validated coaching model during the VBC sessions. Two studies by Singh et al. and Soucisse et al. employed the goals, reality, options, and wrap‐up (GROW) model of coaching, which is a four‐step process that is widely used in business and management [13, 14, 15]. Bonrath et al. adopted a modified PRACTICE model of coaching, which is a seven‐step model developed by Professor of coaching psychology Stephen Palmer [16, 17].

**TABLE 2:** Coaching strategies and scoring systems.

Author (Year)	Control			VBC					Technical assessment
	*n*	Feedback	No. of sessions	*n*	Session length (mins)	Timing of coaching	No. of sessions	Validated coaching model	
Backstein (2004)	29	No specific feedback	N/A	29	15	Immediate	1	N/A	OSATS (GRS, TSC) time
Backstein (2005)	12	Practice sessions with feedback from circulating experts as part of the normal course	N/A	14	15	Immediate	3	N/A	miniOSATS (GRS, TSC)
Heiland (2014)	7	Standard residency curriculum	N/A	5	Not specified	1 week postprocedure	Not specified	N/A	“Single quality‐score sheet”
Bonrath (2015)	9	Standard residency curriculum	N/A	9	Not specified	N/A	4	Modified PRACTICE model	OSATS (GRS), BOSATS, GERT
Nesbitt (2015)	11	20 min PowerPoint presentation covering common errors	1	11	20	Immediate	1	N/A	OSATS (GRS, TSC), pass/Fail
Karam (2015)	7	No specific feedback	N/A	8	Not specified	Not reported	1	N/A	OSATS (GRS) time
Singh (2015)	10	Online surgical lectures	5	10	30	Immediate	5	GROW	OSATS (GRS), GOALS, OPRS time
Abbott (2017)	11	Access to training video 2 weeks before task repetition and 24 h access to simulation center	2	10	Not specified	2 weeks postprocedure	2	N/A	“Performance score”
Phillips (2017)	35	20 min unsupervised review of own performance and review of an expert video	1	36	20	Unclear	1	N/A	TSC, pass/fail
Rindos (2017)	9	Standard residency curriculum	N/A	11	15	Unclear	3	N/A	GOALS
Soucisse (2017)	14	Standard residency curriculum	N/A	14	30	Unclear	1	GROW	OSATS (GRS)
Alameddine (2018)	8	No specific feedback	N/A	8	10	Immediate	1	N/A	Technical skills score (modified GOALS)
Tanaka (2018)	8	No specific feedback	N/A	8	30	Unclear	Not specified	N/A	GOALS‐GH, VAS time
Dickerson (2019)	22	Coaching session with supervising surgeon	1	20	Not specified	Immediate	1	N/A	OSATS (TSC, GRS),
Liao (2020)	8	Conventional perioperative feedback	10	8	45	Within 1 week of procedure	10	Standardized coaching model^a^	GOALS, modified OSATS (TSCh)

^a^ Specific details on coaching model not provided.

### 3.4 Control Groups

Similarly, the control groups were exposed to different forms of feedback across the 15 studies. These feedback methods are summarized in Table 2. Nesbitt et al. and Backstein et al. compared VBC to two groups, each exposed to a different form of feedback, one of which included participants receiving a video of their own performance without individualized feedback from a coach [18, 19]. The remaining 13 studies compared VBC to a single control group, with only Phillips et al. providing the control group with a video of their performance [20].

### 3.5 Technical Performance Assessment

Several different methods of assessing technical performance were used. The objective structured assessment of technical skills (OSATS) was the most frequently used scoring system, with a version of it used in 10 studies. The full breakdown of the scoring systems used are summarized in Table 2.

### 3.6 Differences in Performance Scores

The VBC groups achieved either higher final performance scores or higher change scores from baseline compared to the control groups in 14 of the 15 included studies, with a statistically significant difference reported in ten [11, 12, 14, 15, 17, 19, 21, 22, 23, 24]. Backstein et al. showed no difference in technical performance for orthopedic residents who were randomized to receive either VBC, an unsupervised video review of their own performance without coaching, or no specific feedback at all when performing three separate simulated fracture repairs in their cross‐over trial [18].

**FIGURE 3:**
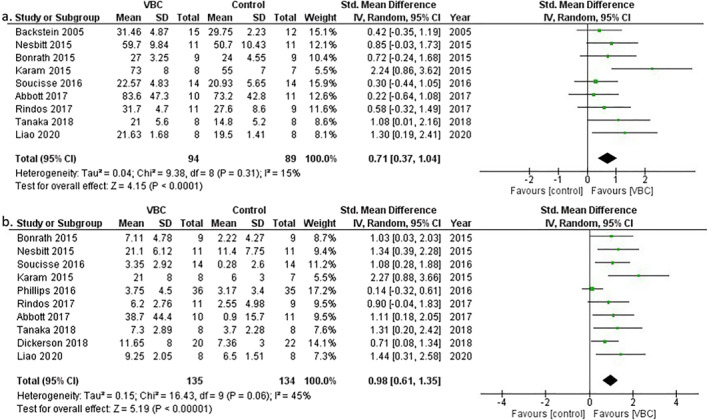
Forest plots of impact of VBC versus control on final performance scores (a) and change from baseline to final scores (b). IV, inverse variance.

A meta‐analysis on nine studies that reported final performance scores was performed [12, 15, 17, 19, 22, 23, 24, 25]. In total, 94 participants received VBC and 89 patients received the control method of feedback. For studies that used multiple objective methods of assessing performance, only the final OSATS score was used within the meta‐analysis as it was the most. Overall, the average performance scores for VBC were significantly higher than the controls, with a SMD of 0.71 (95% CI 0.37, 1.04, *Z* = 4.15, *p* < 0.0001, and Figure 3).

The impact of VBC on change in performance over the study duration was reported in 14 studies with 13 of these reporting a greater improvement in performance for the participants receiving VBC compared to the control groups [11, 12, 14, 15, 17, 19, 20, 21, 22, 23, 24, 26, 27]. Sufficient data were available to perform a meta‐analysis on 10 of these studies [12, 15, 17, 19, 20, 21, 22, 23, 24, 27]. In total, 137 and 132 participants received either VBC or the control method of feedback, respectively. The overall change in performance from the baseline to final assessment was significantly higher in the VBC group compared to the control, with a SMD of 0.98 (95% CI 0.61, 1.35, *Z* = 5.19, *p* < 0.0001, and Figure 3).

The number of feedback sessions received, the coaching model used (previously validated model vs. an unspecified coaching model), the type of surgical task performed (simulated vs. live surgery), and the type of feedback method used in the different control arms of the studies are all potential confounding factors that could impact the summary effect measure. Therefore, these factors were assessed on multiple subgroup analyses (see Supporting Information S1: for figures). No significant differences in final performance scores or change scores were seen whether trainees received single or multiple VBC sessions, or whether the surgical task was performed in a simulated setting or during live surgery, with significantly better scores observed in the VBC groups compared to the controls in all these scenarios (Supporting Information S1: Figures S1 and S2). A further subgroup analysis was performed comparing the use of a previously validated coaching model for participants receiving VBC did not result in significantly higher mean final performance scores (SMD 0.46, 95% CI −0.13, 1.05, *Z* = 1.53, *p* = 0.13, and Supporting Information S1: Figure S3), though significantly higher mean change scores were seen (SMD 1.06, 95% CI 0.43, 1.68, *Z* = 3.32, and *p* < 0.0001, Supporting Information S1: Figure S4). Finally, the different types of feedback received by the control groups were compared. Studies were divided into three categories: those who received no specific feedback; those who received individualized feedback without the video review or access to supplementary material; and those who received supplementary material such as access to training videos or lecture slides. Significantly higher mean performance scores (SMD 1.58, 95% CI 0.45, 2.70, and *p* = 0.006) and mean change scores (SMD 1.26, 95% CI 0.40, 2.11, and *p* = 0.004) were seen in the VBC compared to controls who received no specific feedback. Similarly, both higher mean scores (SMD 0.58, 95% CI 0.19, 0.97, and *p* = 0.004) and mean change scores (SMD 1.08, 95% CI 0.61, 1.56, and *p* < 0.0001) were seen in the VBC group compared to controls who received individualized feedback alone. Although higher scores were also seen in the VBC group when compared to controls who received some form of supplementary training material, these differences did not reach statistical significance (Supporting Information S1: Figures S5 and S6).

## 4 Discussion

Although the need to supplement the conventional apprenticeship model of surgical training has been clear for some time now, the optimum approach remains unknown. Although simulation‐based training for both technical and nontechnical skills has become quite popular, novel training methods, such as VBC, have yet to gain wide‐spread use. In this review, we have demonstrated both a growing interest in the use of VBC and a growing body of evidence to support its use in supplementing conventional training.

Backstein et al. performed the first RCT to investigate the role of individualized VBC on surgical technical skill acquisition in 2004. They compared VBC to two groups, one who received a video of their own performance without additional coaching and one who received no feedback. Participants performed three simulated orthopedic procedures and were exposed to all three feedback methods in a cross‐over trial. The authors found no difference in final performance scores or change in mean performance scores between the three groups across the three procedures [18]. The authors hypothesized that the inclusion of participants with differing degrees of orthopedic experience was likely responsible for the lack of difference seen across feedback methods. Therefore, they repeated a VBC study in 2005 in which participants from a range of surgical specialties were exposed to either VBC or standard verbal feedback while performing a benchtop vascular anastomosis. Although the VBC group achieved higher mean performance scores compared to the control, this difference did not reach statistical significance. However, baseline performance scores were not assessed and instead self‐reported experience at performing an intraoperative vascular anastomosis was used to control for baseline differences between the two groups. As such, the impact of VBC on technical skill acquisition or performance improvement could not be reliably assessed [25].

Three more studies reported either higher final performance scores or change scores for VBC but failed to reach statistical significance [20, 26, 27]. Both Alameddine and Phillips et al. assessed the impact of VBC of technical skill acquisition in medical students performing skin suturing in a simulated setting [20, 26]. Alameddine et al. performed a pilot study with 16 participants. Those in the VBC group received a single 10 min coaching session in which they reviewed a video of their own performance with expert feedback, whereas those in the control group did not receive any specific feedback. On task repetition, total change scores where actually higher in the control group than the VBC on the independent faculty assessment, whereas on self‐assessment, the VBC recorded higher change scores than the control group, though neither achieved statistical significance [26]. Phillips et al. performed a larger study of 71 participants, in which the VBC group received a single 20 min coaching session, whereas the control group received 20 min to review a video of their own performance without any expert feedback. Higher change scores were reported for the VBC group, though this did not reach statistical significance [20]. Finally, Dickerson et al. compared a single VBC session immediately after performing a simulated distal tibial fracture repair to a single coaching session without video review. Higher change scores were reported in the VBC group compared to the control without achieving statistical significance [27]. There are a few potential explanations for lack of significant differences between VBC and the control groups in these studies. Neither Alameddine et al. nor Phillips et al. reported baseline performance scores for the two groups, and therefore, it is possible that baseline differences between the groups could explain the lack of significant differences seen in final performance. Furthermore, the ten‐minute VBC session used by Alameddine et al. was the shortest reported coaching session of any of the included studies, and so it is possible that longer sessions are needed to see a meaningful difference in performance.

Indeed, although the remaining 10 studies demonstrated statistically significant higher scores for VBC, the optimum length of VBC sessions remains unclear. Out of the included studies, session length ranged from 10 minutes to 45 min. Only one of the six studies that included VBC sessions of 20 min or longer failed to show a statistically significant benefit of VBC to the control, whereas three of the four studies that used shorter than 20 min sessions failed to show a significant difference. Therefore, it is likely that the optimum session length is over 20 min, but further studies are required to confirm this. Similarly, the optimum timing of VBC sessions requires further investigation, though based on the subgroup analyses performed in this review it appears to be effective whether performed immediately after or within 2 weeks of the procedure.

The results of this study are in keeping with the results of two previously performed systematic reviews. Augestad et al. published the first and largest review of VBC to date, including 24 RCTs and 778 participants. The studies included RCTs that compared VBC to various control methods of feedback in both simulated and live surgeries and included both medical students and surgical residents within the study populations. The authors reported higher performance scores for VBC compared to controls on meta‐analysis (SMD 0.87, SD 0.46–1.09, and *p* < 0.001) [7]. However, measuring final performance scores alone introduces several confounders, particularly when baseline performance has not been measured. Although many of the RCTs in the review by Augestad et al. attempted to control for this by including participants of similar experience levels, potential differences could remain. To account for this, in our review, meta‐analyses of both final performance scores and change scores were performed. When change scores were used, the effect size in favor of VBC was always larger than when final scores alone were compared, suggesting that the benefit of VBC was likely underestimated in the RCTs that did not control for differences in baseline performance. Daniel et al. performed an updated meta‐analysis of VBC in surgical residents only. In total, 11 RCTs, including 298 participants, were identified and included four studies that did not feature in the review by Augestad et al. As in our review, the authors reported differences in both final performance scores and change scores. They found no significant difference between VBC and the controls in final performance scores but significantly higher change scores for VBC (SMD 1.62, 95% CI 0.62, 2.63, *p* = 0.002) [8]. However, both prior systematic reviews used a much broader definition of VBC than this study. Although there is no universally accepted definition of VBC, the authors believe that the definition used in this study better reflects what is generally accepted as VBC.

Although the results of this study have clearly shown a benefit to VBC, there are of course several limitations to discuss. The included studies were of relatively small sample size, with only one study including over 50 participants. Although the meta‐analyses performed revealed only a low to moderate degree of statistical heterogeneity, there is significant heterogeneity between the included studies in terms of study participants, the surgical task performed, the assessment method, and the control method of feedback. Therefore, multiple subgroup analyses were performed to minimize the impact of these differences, and the results have confirmed VBC to be effective in both simulated and live surgeries, whether delivered as a single session or over multiple sessions and across a wide range of coaching frameworks, proving it to be a versatile training tool. Perhaps, the most encouraging finding was that VBC improved technical performance whether a validated coaching framework was used or not, meaning that it can be used by all surgical trainers and not just those who have been previously trained in a defined coaching model. Finally, although the benefit of VBC has been clearly demonstrated across a range of surgical subspecialties and in both open and laparoscopic surgeries alike, further research is needed to establish how best it can be incorporated into surgical training curricula going forward.

## 5 Conclusion

This systematic review represents the most comprehensive review of VBC in surgical training to date. The results confirm that VBC is a highly effective and versatile method of improving surgical technical skill acquisition when compared to several different forms of feedback that are routinely used in surgical training to date. In an era where the traditional apprenticeship model of surgery has become threatened by reduced working hours and surgical volume, VBC has emerged as an appealing method of bridging the gap that currently exists in surgical training.

## Author Contributions

**Sarahlouise Gillanders:** data curation, methodology, writing–original draft. **Anne Hickey:** writing–review & editing. **Gozie Offiah:** writing–review & editing. **Niall Davis:** writing–review & editing.

## Conflicts of Interest

The authors declare no conflicts of interest.

## Supporting information

Supporting Information S1

## Data Availability

The data that support the findings of this study are not publicly available but may be available upon request from the corresponding author.
