# Supplementary material for: Video Killed the Radio Star—A Meta‐Analysis on Video‐Based Coaching to Improve Surgical Skill
Source: World J Surg. 2025 Feb 12;49(3):634–42. doi: 10.1002/wjs.12494 (PMC11903253; doi:10.1002/wjs.12494)
Supplement: Supplementary file 1 — Supporting Information S1 [file WJS-49-634-s001.docx]

**Supplementary material:**

**ELIGIBILTY CRITERIA**

***Inclusion criteria***

Studies assessing the impact of VBC on surgical technical skills performance were assessed for eligibility based on the following inclusion criteria:

1. Study design:
   1. Randomised controlled trials
2. Participants:
   1. Undergraduates - medical students
   2. Postgraduates - surgical trainees or consultants (in any surgical sub-specialty)
3. Index procedure:
   1. Any clinical, technical skill in surgery (suturing, laparoscopic procedures etc.)
4. Intervention:
   1. Participants received video review of their own performance with an expert providing feedback/coaching
5. Outcome:
   1. Quantitative measure of technical performance

***Exclusion criteria***

Studies were excluded if any of the following applied:

1. Study design:
   1. No comparison group, such as controlled before and after studies – this was to avoid the potential confounding variable of simple task repetition resulting in improved performance
2. Participants:
   1. Undergraduates – non-medical students
   2. Postgraduates - non-surgical trainees or consultants who perform procedures (cardiologists, gastroenterologists, radiologists etc.)
3. Index procedure:
   1. Non-technical tasks (communication skills, leadership skills etc.)
   2. Non-surgical technical skills (intravenous cannulation, phlebotomy, catheterisation etc.)
4. Intervention:
   1. No video review of own performance
   2. No expert providing feedback during video review
5. Outcome:
   1. Qualitative measures only

**STATISTICAL ANALYSIS**

There was significant heterogeneity in the reporting of outcome measures across the included studies. As a result, not all studies reported outcomes that facilitated a quantitative pooling of data. For these studies that did not report outcomes in a method that allowed SMD to be calculated, the raw data was requested from the authors. If the raw data was not available, or if the authors did not respond, the results from these studies were omitted from the meta-analyses. For studies reporting differences in baseline to final performance scores (change scores) without reporting a SD, the SD was either calculated from the 95% confidence intervals or imputed using a correlation coefficient or the SD from a closely related study.

**SUBGROUP ANALYSES**
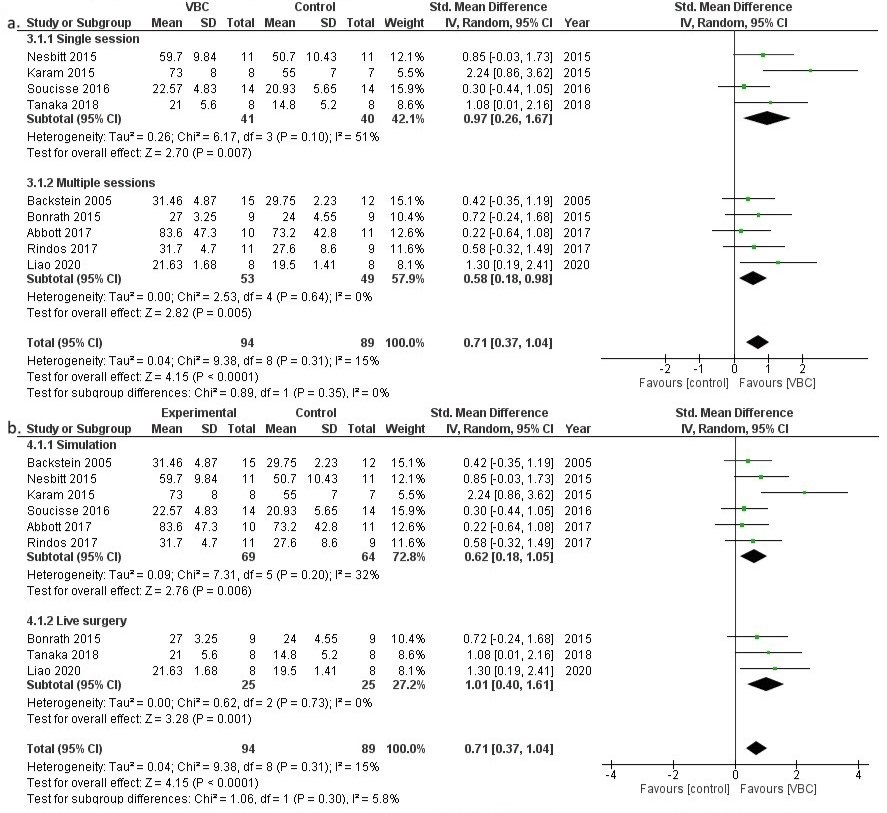


**Figure S1 Subgroup analyses comparing the effects of the number of VBC sessions (a.) and the types of surgical procedures performed (b.) on final performance scores**


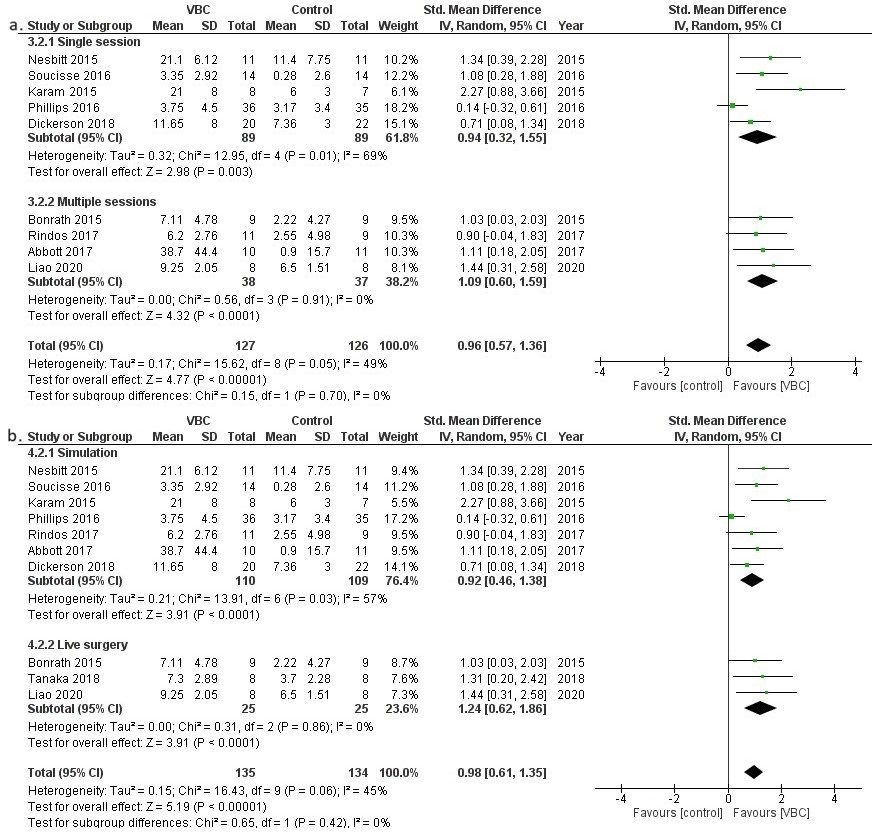


**Figure S2 Subgroup analyses comparing the effects of the number of VBC sessions (a.) and the types of surgical procedures performed (b.) on change scores**


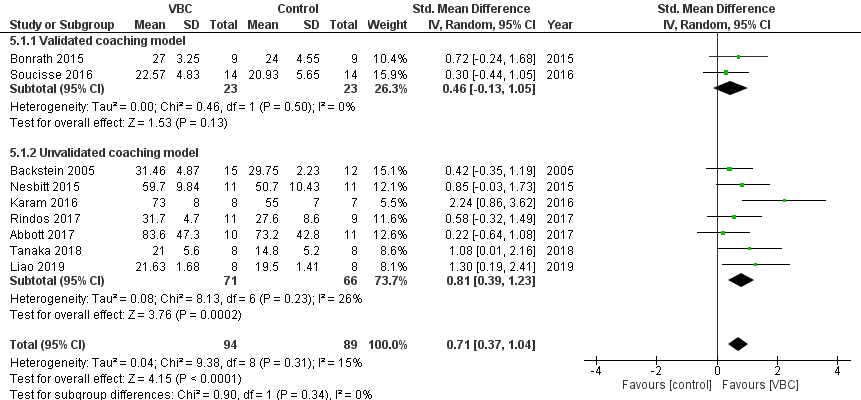


**Figure S3 Subgroup analysis comparing the effects of the coaching model used in the VBC groups on final performance scores**


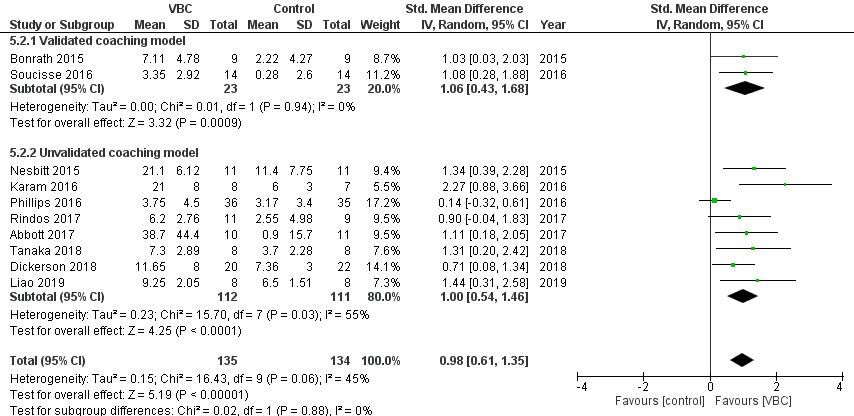


**Figure S4 Subgroup analysis comparing the effects of the coaching model used in the VBC groups on change scores**

**
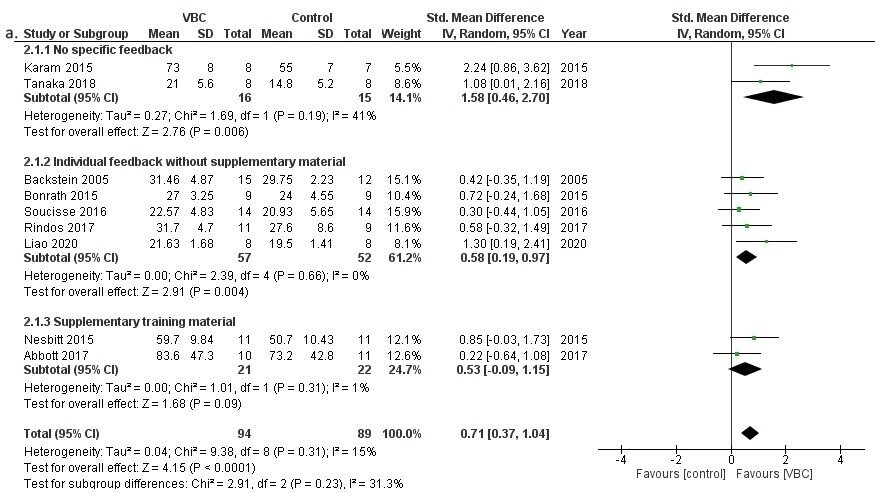
**

**Figure S5 Subgroup analysis comparing the different types of feedback received by the control arms on final performance scores**

**
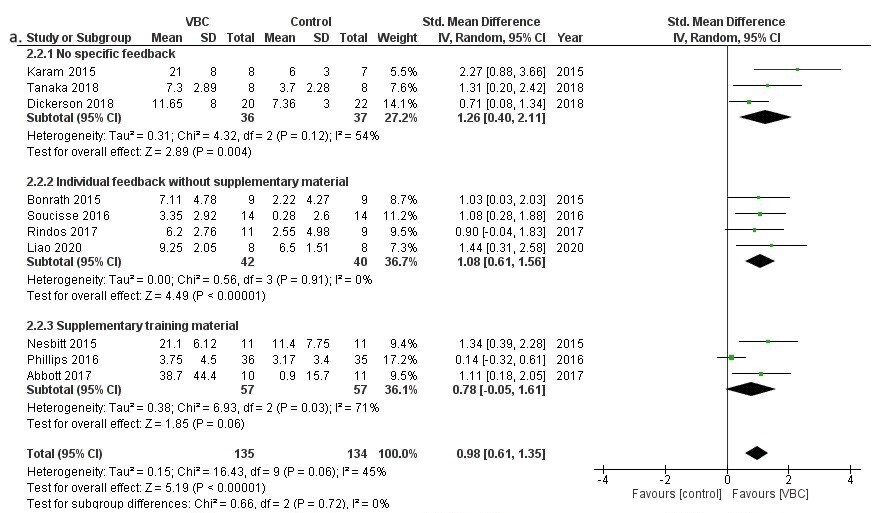
**

**Figure S6 Subgroup analysis comparing the different types of feedback received by the control arms on change scores**

**SECONDARY OUTCOME MEASURES:**

*Time to task completion*

Only four studies reported the impact of VBC on time to task completion (TTC), and there was insufficient data reported to perform a meta-analysis [14, 18, 21, 24]. Singh et al. reported significantly longer TTC for the VBC group compared to the control group when performing their final porcine laparoscopic cholecystectomy in their study (2297 vs 1683 seconds, p=0.003) [14]. The remaining three studies reported shorter TTC for the VBC groups, but these differences did not reach statistical significance [18, 21, 24].

*Perceived usefulness*

A total of six studies reported the perceived usefulness of VBC as a training tool [15, 17, 21, 25-27]. In five of these studies, VBC was reported to be a very useful training tool by either all the participants or an overwhelming majority of the participants [15, 17, 21, 26, 27]. Backstein et al. did not provide a global summary of the perceived usefulness of VBC as reported by the study participants, instead the authors reported that some participants felt VBC “allowed them to fine-tune a basic skill” while others reported that VBC may be more useful once trainees had already “acquired the basic skill level for the task” [25].
